# Real-life effectiveness of a new allergic rhinitis therapy (MP29-02*) in Sweden

**DOI:** 10.1186/2045-7022-5-S4-P37

**Published:** 2015-06-26

**Authors:** Pär Stjärne, Victoria Strand, Kaj Theman, Hans Christian Kuhl, Anders Ehnhage

**Affiliations:** 1 Karolinska Institute, Dept of Clinical Science, Stockholm, Sweden; 2 Astma och Allergimottagningen, Stockholm, Sweden; 3 Astma och Allergimottagningen, Göteborg, Sweden; 4 Meda, Bad Homburg, Germany

## Background

Over one quarter of individuals in Sweden report suffering from allergic rhinitis (AR), placing a considerable burden on both sufferers and society [1]. In clinical trials MP29-02* (a novel intranasal formulation of azelastine hydrochloride (AZE) and fluticasone propionate (FP) in an advanced delivery system) provided complete/near complete symptom control in 1 of 6 moderate/severe seasonal AR (SAR) patients [2] and complete relief in 7 of 10 mild/moderate perennial AR (PAR) patients [3]. This study aimed to assess the effectiveness of MP29-02* in routine clinical practice.

## Method

Results from Sweden (n=431) from a multinational, multicentre, prospective, observational study in adults/adolescents with moderate/severe AR for whom MP29-02* was prescribed according to SPC are reported. Patients had acute AR symptoms on Day 0. Intended study duration was 14 days. Patients assessed symptom severity using a visual analog score (VAS) from 0mm (not at all bothersome) to 100mm (very bothersome), in the AM prior to MP29-02* use, on Days 0, 1, 3, 7 and last day. This was described for the whole population and according to phenotype (i.e. SAR, PAR or SAR + PAR) and severity (less severe: baseline VAS=50-74mm; more severe: baseline VAS ≥75mm). Patients’ perceived level of disease control (i.e. well-, partly- and un-controlled) was assessed on Day 3.

## Results

MP29-02* (1 spray/nostril bd; daily doses: AZE:548µg, FP:200µg) reduced VAS score from 67.9mm at baseline to 32.1mm by last visit, a shift of 36.1mm. Effectiveness was consistent regardless of phenotype or disease severity. Symptom burden reduced rapidly in the first days of treatment. 27% of all patients felt their symptoms were ‘well controlled’ and 43% felt their symptoms were ‘partly-controlled’ at Day 3. This perception of ‘well-controlled’ symptoms at Day 3 corresponded to an optimal VAS cut-off in Sweden of 39mm. On average patients treated with MP29-02* crossed this well-controlled VAS cut-off by Day 7.

## Conclusion

MP29-02* provides effective and rapid symptom control in Swedish AR patients in a real-world setting irrespective of disease phenotype or severity, with responder rates higher than those observed in a clinical trial with moderate/severe AR patients, supporting MP29-02*’s position as the drug of choice for the treatment of AR.

*Dymista
